# Supplementary figures and images for: Predicted versus CT-derived total lung volume in a general population: The ImaLife study
Source: PLoS One. 2023 Jun 16;18(6):e0287383. doi: 10.1371/journal.pone.0287383 (PMC10275439; doi:10.1371/journal.pone.0287383)

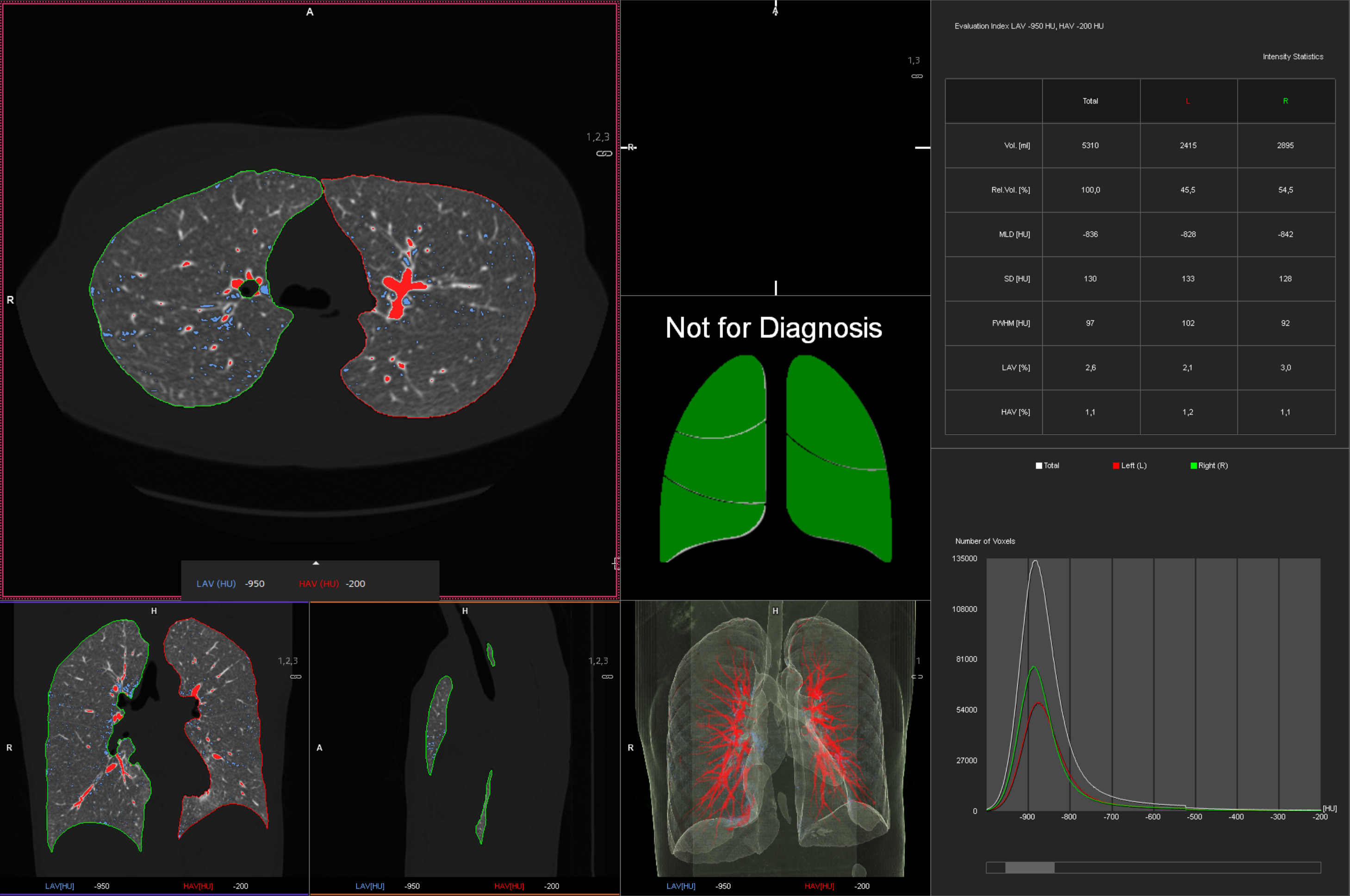

Supplement: S1 Fig — (PNG) [file pone.0287383.s001.png]
