# Supplementary material for: Predicted versus CT-derived total lung volume in a general population: The ImaLife study
Source: PLoS One. 2023 Jun 16;18(6):e0287383. doi: 10.1371/journal.pone.0287383 (PMC10275439; doi:10.1371/journal.pone.0287383)
Supplement: S1 Table — Participants were excluded from the HNS (healthy never-smokers) sub-cohort if they reported a smoking history, had self-reported lung disease, were determined by the spirometry to have COPD, or if any of these were missing. (DOCX) [file pone.0287383.s002.docx]

S1 Table – Population characteristics stratified by sex.

Participants were excluded from the HNS (healthy never-smokers) sub-cohort if they reported a smoking history, had self-reported lung disease, were determined by the spirometry to have COPD, or if any of these were missing.

|  | General population  (cohort GP) | | Healthy participants  (cohort H) | | Healthy never-smokers  (cohort HNS) | |
| --- | --- | --- | --- | --- | --- | --- |
| Variable | Women (N=200) | Men (N=200) | Women (N=142) | Men (N=131) | Women (N=61) | Men (N=58) |
| Age (years) | 54 ± 5.5 | 54 ± 5.4 | 54 ± 5.4 | 53 ± 5.5 | 53 ± 5.4 | 53 ± 5.5 |
| Weight (kg) | 74 ± 12 | 86 ± 10 | 74 ± 12 | 87 ± 11 | 73 ± 13 | 83 ± 11 |
| Height (m) | 1.70 ± 0.07 | 1.83 ± 0.07 | 1.70 ± 0.07 | 1.84 ± 0.07 | 1.70 ± 0.08 | 1.84 ± 0.07 |
| Body-mass index (kg/m²) | 25.6 ± 4.1 | 25.7 ± 2.9 | 25.6 ± 3.8 | 25.7 ± 2.9 | 25.4 ± 4.0 | 24.7 ± 3.0 |
| Smoking status | Never: 84 (42%) Past: 78 (39%) Current: 33 (17%) Missing: 5 (3%) | Never: 85 (43%) Past: 73 (37%) Current: 35 (18%) Missing: 7 (4%) | Never: 61 (43%) Past: 57 (40%) Current: 20 (14%) Missing: 4 (3%) | Never: 58 (44%) Past: 51 (39%) Current: 17 (13%) Missing: 5 (4%) | Never: 61 (100%) Past: 0 (0%) Current: 0 (0%) Missing: 0 (0%) | Never: 58 (100%) Past: 0 (0%) Current: 0 (0%) Missing: 0 (0%) |
| Pack-years (current/past smokers) | 9.1 ± 9.4 | 9.8 ± 8.4 | 7.7 ± 7.4 | 8.9 ± 7.7 | 0 | 0 |
| CT-diagnosed emphysema | None (<5%): 131 (66%) Trace (5-15%): 68 (34%) Mild (>15%): 1 (1%) | None (<5%): 80 (40%) Trace (5-15%): 107 (54%) Mild (>15%): 13 (7%) | None (<5%): 98 (69%) Trace (5-15%): 43 (30%) Mild (>15%): 1 (1%) | None (<5%): 57 (44%) Trace (5-15%): 70 (53%) Mild (>15%): 4 (3%) | None (<5%): 43 (71%) Trace (5-15%): 18 (30%) Mild (>15%): 0 (0%) | None (<5%): 29 (50%) Trace (5-15%): 28 (48%) Mild (>15%): 1 (2%) |
| Forced expiratory volume in 1 second (FEV₁, L) | 2.9 ± 0.5 | 4.0 ± 0.7 | 2.9 ± 0.5 | 4.2 ± 0.6 | 2.9 ± 0.5 | 4.2 ± 0.6 |
| Forced vital capacity (FVC, L) | 3.9 ± 0.7 | 5.4 ± 0.8 | 3.8 ± 0.6 | 5.3 ± 0.8 | 3.8 ± 0.7 | 5.4 ± 0.7 |
| GOLD stage | I: 20 (10%) II: 8 (4%) III: 0 (0%) IV: 0 (0%) | I: 32 (16%) II: 13 (7%) III: 1 (1%) IV: 0 (0%) | I: 0 (0%) II: 0 (0%) III: 0 (0%) IV: 0 (0%) | I: 0 (0%) II: 0 (0%) III: 0 (0%) IV: 0 (0%) | I: 0 (0%) II: 0 (0%) III: 0 (0%) IV: 0 (0%) | I: 0 (0%) II: 0 (0%) III: 0 (0%) IV: 0 (0%) |
| Self-reported lung disease | 19 (10%) | 24 (12%) | 0 (0%) | 0 (0%) | 0 (0%) | 0 (0%) |

Values are mean±SD or N (percentage).
