# Supplementary material for: Predicted versus CT-derived total lung volume in a general population: The ImaLife study
Source: PLoS One. 2023 Jun 16;18(6):e0287383. doi: 10.1371/journal.pone.0287383 (PMC10275439; doi:10.1371/journal.pone.0287383)
Supplement: S2 Table — (DOCX) [file pone.0287383.s003.docx]

S2 Table – outcome comparison

|  | General population (N=200+200) | Healthy participants (N=142+131) | Healthy never-smokers (N=61+58) |
| --- | --- | --- | --- |
| Systematic bias (L) | F: 0.9 (p=0.388) M: 1.4 (p=0.094) | F: 1.0 (ref) M: 1.7 (ref) | F: 1.0 (p=0.556) M: 1.8 (p=0.591) |
| ΔLoA (L) | F: 3.1 (p=0.778) M: 4.6 (p=0.259) | F: 3.2 (ref) M: 4.2 (ref) | F: 3.4 (p=0.377) M: 4.0 (p=0.784) |

F: women, M: men, ΔLoA: difference between the 95% limits of agreement
