# Supplementary material for: Predicted versus CT-derived total lung volume in a general population: The ImaLife study
Source: PLoS One. 2023 Jun 16;18(6):e0287383. doi: 10.1371/journal.pone.0287383 (PMC10275439; doi:10.1371/journal.pone.0287383)
Supplement: S1 Equations — (DOCX) [file pone.0287383.s004.docx]

S1 Equations – Results of linear regression analysis.

An optimized linear regression model based on the study population resulted in the following prediction formulae of lung volume (in L) for women (V_F_) and men (V_M_):

$$V_{F}= -7.309+7.442*H-0.010*W+0.003*A$$

$$V_{M}=-9.281+9.229*H-0.032*W+0.023*A$$
